# Sex-Specific Brain Transcriptional Signatures in Human MDD and Their Correlates in Mouse Models of Depression

**DOI:** 10.3389/fnbeh.2022.845491

**Published:** 2022-05-03

**Authors:** Maureen Touchant, Benoit Labonté

**Affiliations:** ^1^CERVO Brain Research Centre, Québec, QC, Canada; ^2^Department of Psychiatry and Neuroscience, Faculty of Medicine, Université Laval, Québec, QC, Canada

**Keywords:** stress, rodents, sexual dimorphism, resilience, susceptibility, behavioral stress responses, transcription profiles/signatures

## Abstract

Major depressive disorder (MDD) is amongst the most devastating psychiatric conditions affecting several millions of people worldwide every year. Despite the importance of this disease and its impact on modern societies, still very little is known about the etiological mechanisms. Treatment strategies have stagnated over the last decades and very little progress has been made to improve the efficiency of current therapeutic approaches. In order to better understand the disease, it is necessary for researchers to use appropriate animal models that reproduce specific aspects of the complex clinical manifestations at the behavioral and molecular levels. Here, we review the current literature describing the use of mouse models to reproduce specific aspects of MDD and anxiety in males and females. We first describe some of the most commonly used mouse models and their capacity to display unique but also shared features relevant to MDD. We then transition toward an integral description, combined with genome-wide transcriptional strategies. The use of these models reveals crucial insights into the molecular programs underlying the expression of stress susceptibility and resilience in a sex-specific fashion. These studies performed on human and mouse tissues establish correlates into the mechanisms mediating the impact of stress and the extent to which different mouse models of chronic stress recapitulate the molecular changes observed in depressed humans. The focus of this review is specifically to highlight the sex differences revealed from different stress paradigms and transcriptional analyses both in human and animal models.

## Introduction

Major depressive disorder (MDD) represents one of the top causes of disability worldwide (Vos et al., 2017). Recent studies estimate that more than 20% of the population worldwide will be affected at least once in their life by depressive episodes which ultimately translates into a major burden on modern societies (Alonso et al., 2004). Despite the importance of the disease, little progress has been made in understanding the etiologies of MDD. However, recent progress with fast-acting antidepressant molecules shows promising perspectives in the treatment of this disorder (Berman et al., 2000; Thelen et al., 2016; Mandal et al., 2019; Polis et al., 2019; Ouyang et al., 2021).

From a clinical perspective, MDD is a highly heterogenous disease defined by complex clinical manifestations including depressed mood or irritability, anhedonia, grief, guilt, apathy, self-injury, indecision, and concentration disorders. This also includes psychomotor retardation, vegetative symptoms with sleep, appetite, and stress hormone dysregulation that is associated with either gain or loss of weight, suicidal ideation, and cognitive disorder (American Psychiatric Association, 2003). These clinical features are expressed and shared by both men and women with MDD despite important sex differences (Weissman and Klerman, 1977; Nolen-Hoeksema, 1987; Salk et al., 2017; Eid et al., 2019). Past and recent epidemiological studies show that the prevalence of MDD is about 20% in a lifetime with a higher incidence in women, and females are two-three times more susceptible than males. Women also exhibit earlier age of onset (Kessler et al., 1993), higher symptom severity from childhood (Kessler et al., 2007; Marcus and Flynn, 2008; McLean et al., 2011; Avenevoli et al., 2015), and higher rates of depressive episodes (Bertschy et al., 2016) than men. At the clinical level, men and women diagnosed with MDD express more or less the same symptoms although their prevalence varies in a sex-specific fashion. For instance, aggression, substance abuse, and risk-taking behaviors are more prevalent in males (Martin et al., 2013), while women with MDD exhibit higher rates of comorbid anxiety (Regier et al., 1990; Kessler et al., 1994; Schuch et al., 2014). A higher prevalence of atypical depression is also observed in women. In men and women, this is defined by the expression of reactive mood to environmental cues, increased appetite, hypersomnia, leaden paralysis, and interpersonal rejection sensitivity. While MDD in women is defined by a higher prevalence of internalized disorders such as ruminating and emotionality, externalized symptoms are more common in men including constraint and aggressive behavior (Krueger et al., 2001).

Crucial insights into the molecular and functional mechanisms underlying differences between males and females with MDD have resulted from studies performed in human populations or post-mortem tissue, some of which have forged our pathophysiological conception of the disease (Rajkowska, 2003; Tham et al., 2011; Zhao et al., 2019). For instance, studies have revealed functional, morphological, and molecular changes affecting the activity of several brain regions in MDD (Frodl et al., 2008; Ramezani et al., 2014; Lu et al., 2016; Li et al., 2021). These studies alone have generally provided limited mechanistic insights into the pathophysiological processes underlying the expression of the disease. Mechanistic insights have also been obtained using animal models of stress or depressive-like behaviors. Indeed, past decades have seen the development of several animal models of stress-induced depressive-like behaviors. These models have mostly been developed based on McKinney and Bunney’s criteria (McKinney and Bunney, 1969) of external validity that was later refined by Willner (Willner, 1984, 1991) as predictive, face, and construct validity. This has led to the development of a wide variety of mouse models based on physical, psychosocial, and/or genetic paradigms, each reproducing common and distinct aspects of stress and anxiety-like behaviors in humans (Deussing, 2006; Abelaira et al., 2013; Planchez et al., 2019).

With technological developments to map transcriptional profiles induced by different types of stress, these models provide unique insights into the transcriptional programs that underly the expression of complex behavioral phenotypes in MDD. Importantly, by combining human and mouse data, these studies are now providing highly translational insights into the morphological and functional impact of stress and the function of the brain while highlighting some of the molecular mechanisms underlying these effects (aan het Rot et al., 2009; Duman and Voleti, 2012; Penninx et al., 2013). However, most of the research on this topic has been performed in males, predominantly leaving females understudied for years. Several of the most widely used mouse models of stress and anxiety were originally developed in males; only very recently have the models been revisited to include female cohorts (Lopez and Bagot, 2021). This will provide new opportunities to better understand the common but also distinct mechanisms underlying the development and expression of anxiety and depressive-like behaviors in males and females.

In this review, we first elaborate on the behavioral features exhibited by mouse models of stress with an emphasis on their respective validity in both males and females. We then discuss the most recent findings generated by genome-wide transcriptional studies in both human and mouse models. We also review the main findings that describe the transcriptional impact of different types of chronic stress in males and females. Along these lines, we draw important parallels with findings from studies in humans with MDD to evaluate the capacity of these models to reproduce the transcriptional signatures associated with the expression of the human disease in a sex-specific fashion.

## Animal Models of Depression

The clinical heterogeneity of MDD and anxiety has always represented a challenge in selecting appropriate mouse models. According to McKinney and Bunney (1969), animal models should mimic the human condition (face validity), be relevant to human pathological mechanisms (construct validity), and demonstrate drug efficacy (predictive validity). In this context: (1) face validity refers to the capacity of a model to reproduce the phenomenological, behavioral, anatomical, or phenotypic properties observed in human patients; (2) construct validity refers to the stress paradigm (psychosocial, physical, etc.) to explain theoretically what humans experience in real life; and (3) predictive validity refers to the capacity of pharmacological or non-pharmacological treatments to rescue anxiety and depressive-like behaviors as it would in humans (Willner, 1984, 1991; McKinney, 2001; Willner and Mitchell, 2002; Nestler and Hyman, 2010). Additional features have since then been included in these criteria including mechanistic (common underlying mechanisms in humans and animals), homological (adequate species and strains), and pathogenic (challenges triggering the expression of the pathological state) validity (Belzung and Lemoine, 2011). In the following section, we start by describing some of the most widely used mouse models of anxiety- and depressive-like behaviors to evaluate their respective capacity to achieve high levels of validity in both males and females.

### Chronic Unpredictable/Variable Stress

Models of stress based on the administration of physical stressors refer to the idea that low levels of chronic and unpredictable physical stress, mimicking daily-life stress exposure in humans, trigger the expression of anxiety and depressive-like behaviors in individuals (Kendler et al., 1998, 1999; Haroon et al., 2012). Indeed, clinical and epidemiological studies report that mild but repeated stressors throughout life increase vulnerability to anxiety and depression in men and women (Kessler et al., 1985). Examples of these models that rely on the repeated administration of physical stressors include chronic mild stress (CMS; Katz et al., 1981; Katz, 1982; Forbes et al., 1996; Willner, 2016), chronic unpredictable stress (CUS; Monteiro et al., 2015), chronic unpredictable mild stress (CUMS; Frisbee et al., 2015; Burstein and Doron, 2018), and chronic variable stress (CVS; Willner et al., 1992; Hodes et al., 2015; Labonté et al., 2017) models. CUMS/CMS involves continuous (6–8 weeks) unpredictable exposure to stressful stimuli including wet cage, damp bedding, bedding removal, cage tilt, alterations of light/dark cycle, shallow water bath, restraint, and predator sounds/smells (Katz et al., 1981; Willner, 2016). CVS involves daily exposure to mild foot shocks, tail suspension, or tube restraint for 3 weeks (LaPlant et al., 2009; Hodes et al., 2014; Labonté et al., 2017). Importantly, each model induces a complex phenotype defined by anxiety, behavioral despair, and anhedonia in both males and females. Additionally, subchronic CVS (sCVS), consisting in exposing mice to 6 days of stress rather than 21 days, has been shown to induce an anxiety and depressive-like phenotype in females but not males, mimicking variations in stress susceptibility in both sexes (Hodes et al., 2015; Fatma and Labonté, 2019). The chronic restraint stress (CRS) has often been used as an alternative to CUMS or CVS. However, the nature of the paradigm, along with the type of behavioral consequences induced by CRS, challenges its construct and face validity criteria. Males seem to respond to CRS in a time-dependent manner (Selye, 1976; Beck and Luine, 1999, 2002; Gomez et al., 2012; Gomez, 2013), which confirms the allostatic load concept (McEwen and Stellar, 1993), while females demonstrate a resilient phenotype (Bowman et al., 2001; Bowman and Kelly, 2012). Chronic treatment with antidepressants reverses these depressive-like phenotypes (Stone et al., 1984; Ulloa et al., 2010; Yu et al., 2012).

### Learned Helplessness

Learned helplessness is a model in which animals are exposed to unpredictable stress, after which they develop behavioral deficits in escaping aversive situations. Subjecting mice to situations in which they have no control (e.g., electroshocks) results in motivational, cognitive, and emotional deficits (Abramson et al., 1978). The behavioral deficits induced by learned helplessness are characterized by anxiety, anhedonia, and behavioral despair in males and females (Caldarone et al., 2000; Anisman and Merali, 2001; Chourbaji et al., 2010) that can be reversed by the administration of fast-acting antidepressants drugs (Ramaker and Dulawa, 2017). Additionally, not all mice in this model display helplessness (22%), with a high percentage (78%) exhibiting resilience regardless of the mice’s sex (Kim et al., 2016), further supporting the face validity. However, it should be noted that controversial aspects restrict its usage (Teasdale, 1978). Indeed, it has been suggested that learned helplessness may rely on the motivation to avoid aversive challenges (Maier et al., 1976; Dweck and Wortman, 1982; Kuhl, 1984), rather than inducing a robust emotional response (Beck, 1967, 1987; Abramson et al., 1989; Rose and Abramson, 1992; Possel and Thomas, 2011; Liu et al., 2015). Even though this model reproduces certain behavioral aspects of anxiety and depression in humans, further validation is required to truly reproduce the emotional responses associated with anxiety and MDD in men and women.

### Social Isolation

Psycho-social stress refers to any situation that threatens the psychological need of being affiliated with others and to maintain social self (Cannon, 1932). This can range from social evaluation of performance achievement to social devaluation such as bullying (Björkqvist, 2001; Silver and Teasdale, 2005; Brunstein Klomek et al., 2007; Nedg et al., 2011; Vinkers et al., 2014). In animals, this concept has been modeled by different approaches but mainly through prolonged social isolation (SI; Panksepp et al., 1991). SI has a high construct validity and is highly relevant to the study of human depression and anxiety disorders (Costello and Kendrick, 2000; Heinrich and Gullone, 2006; Wallace et al., 2009). SI also achieves good face validity from a behavioral perspective. For instance, losing a partner or chronic SI induces the expression of depressive-like behaviors in monogamous prairie voles, notably anhedonia, with females being more sensitive to isolation (Grippo et al., 2007). Prolonged SI also induces sex-specific depressive and anxiety-like behaviors such as despair, compulsive and obsessive behaviors, and cognitive defects in a wide range of species including mice, rats, flies, birds, and monkeys (Mercier et al., 2003; Cacioppo et al., 2006; Nonogaki et al., 2007; Apfelbeck and Raess, 2008; Cacioppo and Hawkley, 2009; Han and Richardson, 2010; Makinodan et al., 2012; Amiri et al., 2014; Hom et al., 2017; Tan et al., 2019; Rogers et al., 2020). Interestingly, rather than inducing social avoidance, socially isolated mice have been reported to interact more with their congeners (Lefebvre et al., 2020). Furthermore, when returned to social groups, the behavioral alterations induced by SI are rapidly rescued by social interactions (Zhao et al., 2021). Nonetheless, several molecular alterations that reproduce the human condition have been reported in socially isolated animals, further supporting the face validity of this model. Yet, most of these studies have been performed in males (Lu et al., 2003; Liu et al., 2012; Siuda et al., 2014; Cole et al., 2015; Ieraci et al., 2016).

### Chronic Social Defeat Stress

The chronic social defeat stress (CSDS) animal model reproduces the context of bullying and excessive competitive behaviors in a social environment. In humans, these stressors are strongly associated with a significant increase in adverse mental health consequences and elevated suicide rates (Meltzer et al., 2011). CSDS involves submitting a mouse, either male or female, to repeated bouts of physical subordination followed by prolonged sensory stressors (odor, vocalization, intimidation) without physical contact (Berton et al., 2006; Golden et al., 2011; Harris et al., 2018). By design, it represents a model combining physical and psychosocial bases. Given that male mice are naturally not aggressive with female congeners, protocol adaptations have been proposed to study the impact of chronic social stress in females. One involves luring the resident male by masking the females’ scent and pheromones (Harris et al., 2018). Consequently, the resident males impose repeated bouts of physical aggression on the intruder females. Another approach involves triggering aggressive behaviors in resident male mice by chemogenetically activating the ventromedial hypothalamus. This results in prolonged aggressive behaviors toward female intruders (Takahashi et al., 2017). Interestingly, male and female mice that endure CSDS develop phenotypes of susceptibility or resilience to social stress. This confirms the high levels of face validity. Susceptibility to social stress in both sexes is defined by the expression of social withdrawal, anhedonia, anxiety, behavioral despair, cognitive impairments, and metabolic alterations (Takahashi et al., 2017; Harris et al., 2018). In contrast, resilient animals do not express social withdrawal nor anhedonia but exhibit anxiety-like behaviors (Krishnan et al., 2007; Golden et al., 2011; Takahashi et al., 2017; Harris et al., 2018). Importantly, susceptibility-related behavioral deficits can be rescued by the administration of conventional and fast-acting antidepressant molecules supporting the predictive validity of this model in both males and females (Hare et al., 2017; Hashimoto, 2019).

It should be noted that susceptibility and resilience to social stress are greatly influenced by the mouse’s genetic background (Goyens and Noirot, 1975; Kudryavtseva and Bakshtanovskaya, 1989; Kudryavtseva, 1994; Fuchs et al., 2001; Berton et al., 2006; Huhman, 2006; Miczek et al., 2008; Golden et al., 2011; Laine et al., 2018). The original CSDS protocol (Berton et al., 2006; Golden et al., 2011) was designed with the C57BL/6J mouse strain and reported a rate of resilience to social stress around 30% to 40% (Berton et al., 2006; Golden et al., 2011). However, studies that compared different inbred mouse strains reported varying proportions, with 23% of BALB/c, 19% of 129, and 5% of D2 mouse strains being resilient to CSDS (Dadomo et al., 2011; Razzoli et al., 2011; Savignac et al., 2011; Laine et al., 2018). Together, this suggests that the genetic background in mice has an important impact on the coping strategies with social stress, and more work should be performed with male and female mice to test whether the same conclusions stand.

### Vicarious Chronic Social Defeat Stress

Interestingly, CSDS paradigm variations are now used to study the impact of witnessing social defeat in mice. The vicarious CSDS model (Warren et al., 2013; Sial et al., 2016; Iñiguez et al., 2018) consists of having mice witnessing conspecifics during repeated bouts of social defeat. As such, it relies on emotional and psychological stressors with an important social component. The model induces a variety of behavioral alterations including decreased social interaction, anxiety, weight loss, and increased corticosterone levels (Warren et al., 2013; Qi et al., 2022) expressed in a transient but also prolonged fashion. Similar to the CSDS model, susceptible and resilient phenotypes are also produced. Antidepressant treatments improved the depressive-like behaviors (Savignac et al., 2011; Yoshioka et al., 2022).

### Social Instability Stress

Another model with a strong psychosocial component is the social instability stress (SIS) model (Schmidt et al., 2008; Green and McCormick, 2013; Scharf et al., 2013; Yohn et al., 2019) where, male and female mice are exposed to unstable social hierarchies every 3 days for 7 weeks, and results in the expression of depressive- and anxiety-like behaviors. Anhedonia is a striking feature of the SIS model while hormonal stress response and novelty response remain unchanged (Dadomo et al., 2011). These effects are reversed by fluoxetine in both sexes (Yohn et al., 2019). This paradigm doesn’t discriminate between resilient and susceptible phenotypes.

### Early-Life Stress

Models such as maternal separation in mice (Plotsky and Meaney, 1993; Meaney, 2001; Millstein and Holmes, 2007) and variations in maternal behavior in rats (Champagne et al., 2003; Brunelli et al., 2015) are also commonly used to reproduce the impact of early-life stress (ELS) on the capacity to deal with stress later in life. In humans, early life trauma, childhood abuse, and parental neglect have significantly been associated with the development of mood disorders (Negele et al., 2015; Lippard and Nemeroff, 2020) in men and women. In rodents, ELS during postnatal development results in lifelong cognitive and emotional alterations that interfere with animals’ ability to react and cope with subsequent stressful events (Everson-Rose et al., 2003). For instance, separated pups are more submissive, and generally seek passive coping strategies later in life (Ménard et al., 2016). Similarly, maternal separation in mice increases the susceptibility to social and physical stress in adulthood in both males and females (Tsuda and Ogawa, 2012; Rana et al., 2015).

Male and female pups raised by mothers that provide low levels of licking and grooming early in life also develop anxious and depressive-like behaviors during adulthood, as opposed to pups raised with high licking and grooming mothers (Liu et al., 1997; Caldji et al., 1998; Zhang et al., 2005). Variations in maternal care can also be induced by either the destruction of the nests or the reduction of nesting material available to the pups (Brunson et al., 2005; Cui et al., 2006; Ivy et al., 2008; Rice et al., 2008). Indeed, these manipulations increase maternal anxiety that trigger deficient and abusive maternal care (Dalle Molle et al., 2012; Murthy and Gould, 2018). Pups raised in these conditions exhibit anxiety- and depressive-like behaviors in adulthood, supporting the translational validity of this approach (Ivy et al., 2008; Wang et al., 2011; Raineki et al., 2012; van der Kooij et al., 2015) although negative results have also been described (Brunson et al., 2005; Rice et al., 2008; van der Kooij et al., 2015).

### Environmental and Genetic Constructs

Models based on genetic considerations are also used to study anxiety and depressive-like behaviors in both sexes. For instance, the *Flinders Sensitive* rat strain displays behavioral changes such as diminished appetite, psychomotor retardation, as well as sleep and immune alterations that resemble specific aspects of clinical MDD attributes in males and females (Overstreet et al., 2005; Dalla et al., 2009; Kokras et al., 2009; Kokras and Dalla, 2014). However, these rats do not exhibit anhedonia, one of the main clinical manifestations of MDD (Overstreet and Wegener, 2013). *Wistar Kyoto* rats are hypertensive and exhibit high anxiety-like behavior in control conditions (Will et al., 2003; McAuley et al., 2009). Males exhibit anhedonia, hypophagia, and weight loss/gain, while these characteristics in females are absent (Burke et al., 2016). Similarly, rats with high (bHR) and low (bLR) levels of exploratory activity in novel environments (Clinton et al., 2011) are used to reproduce aspects of internalizing and externalizing behaviors associated with psychiatric conditions such as anxiety and MDD. High responder rats (bHR) are often highly exploratory, disinhibited, hyperactive and aggressive while low responders (bLR) exhibit hypo-locomotion, anxiety, and depressive-like behaviors to novelty (Stead et al., 2006; Flagel et al., 2010, 2014; Stedenfeld et al., 2011; Prater et al., 2017; Birt et al., 2021). Importantly, these features in both strains begin in early developmental phases, supporting both the construct and face validity of this model. However, as for most models, the majority of studies performed with these rat lines have been accomplished in males.

**Figure 1:**
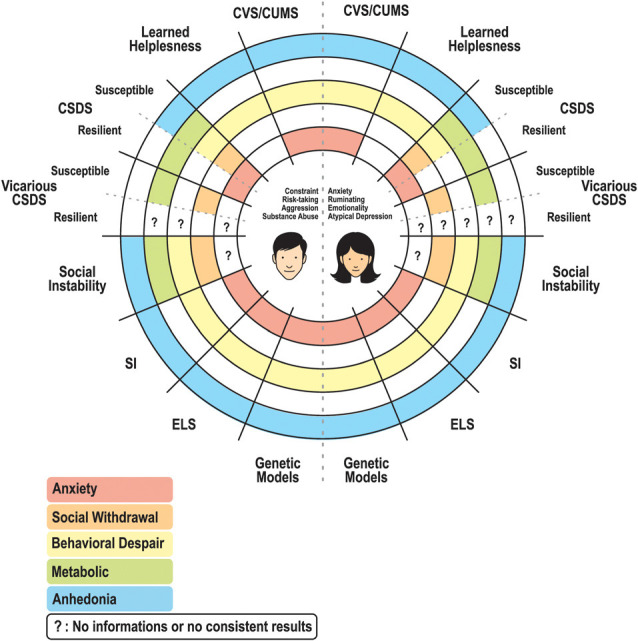
Schematic representation of a circle diagram regrouping the main symptoms (anxiety, social withdrawal, behavioral despair, metabolic dysregulation, and anhedonia) characterizing stress responses in a variety of animal models in males and females. Some unknown results remain, particularly for the resilient group of the vicarious CSDS model, as well as the controversial results concerning the anxiety displayed or not in the social instability model. Abbreviations: CVS/CUMS, chronic variable stress/chronic unpredictable mild stress; CSDS, chronic social defeat stress; SI, social isolation; ELS, early life stress.

Overall, these models support the idea that distinct stress types induce common behavioral phenotypes but also distinct behavioral responses (i.e., social withdrawal, anhedonia, behavioral despair, etc.; Figure 1). It also suggests that no single mouse model can reproduce the full complexity of anxiety and MDD conditions in humans. Rather, one should consider using a model to reproduce one specific aspect, symptom, and/or clinical manifestation of the disease. One also needs to know if these models can reproduce the molecular and transcriptional alterations associated with the human condition. In the next section, we elaborate on the capacity of these models to reproduce not only some of the behavioral features relevant to the disease in humans, but also the transcriptional alterations affecting the brain of men and women suffering from anxiety and depression.

## Sex-Specific Molecular Alterations in MDD

In addition to its capacity to reproduce behavioral features relevant to a human condition, a model’s face validity also relates to its ability to replicate the molecular alterations associated with the disease. This important aspect has been investigated by several groups over the past years, most often using gene candidate approaches (Fatma and Labonté, 2019). Historically, this strategy has been mostly applied to the study of males. Nevertheless, there has been a recent interest in the identification of molecular mechanisms that could underly some aspects of the sexual differences in the expression of anxiety and MDD in men and women. With the availability of genome-wide approaches, combined with the development of highly comprehensive computational strategies, recent studies revealed the transcriptional structures that define stress responses.

### Transcriptional Studies in Human Post-mortem Tissue

Global analyses of the male transcriptome in MDD have revealed several gene-related alterations to different pathways including the glutamatergic, GABAergic, serotonergic, and polyaminergic systems across several cortical and subcortical brain regions (Choudary et al., 2005; Sequeira et al., 2007, 2009, 2012; Klempan et al., 2009; Bernard et al., 2011; Duric et al., 2013). Other studies of cortical regions reported alterations in lipid metabolism, immune response, ATP synthesis, regulation of transcription and translation, fibroblast growth factor signaling, and cell proliferation (Evans et al., 2004; Iwamoto et al., 2004; Kang et al., 2007; Tochigi et al., 2008; Klempan et al., 2009; Lalovic et al., 2010). Furthermore, changes in the regulation of the hypothalamic–pituitary–adrenal (HPA) axis and in the control of circadian rhythms have been reported in the hypothalamus (Wang et al., 2008) and cortical/subcortical regions (Li et al., 2013). However, fewer studies have assessed female transcriptional regulation in MDD. The large majority of these studies adopted a candidate gene approach, showing alterations in brain-derived neurotrophic factor (BDNF), GABAergic, somatostatin (SST), cholinergic, serotonergic, and glutamatergic systems as well as alterations in mitochondrial, energy metabolism, and circadian rhythms in cortical and limbic regions (Boldrini et al., 2008; Szewczyk et al., 2009; Goswami et al., 2010; Lin et al., 2011; Guilloux et al., 2012; Tripp et al., 2012; Bassi et al., 2015; Gray et al., 2015; Seney et al., 2015).

Unfortunately, very few studies directly compared male and female transcriptional profiles. This leaves little comprehension of the molecular mechanisms underlying the expression of the disease in both sexes. The extent to which transcriptional signatures differ between males and females in MDD was assessed by a series of studies published recently. Using RNAseq, Labonté et al. (2017) compared transcriptional signatures across six post-mortem brain regions from men and women with MDD reporting roughly 5%–10% of genes differentially expressed in males and females across all six brain regions. Not only was there a small overlap reported between men and women with MDD, but the directionality of the effects was often opposite in different brain regions. A similar lack of overlap was reported in independent studies also performed on post-mortem brain samples from men and women with MDD (Seney et al., 2018; Girgenti et al., 2021). More recently, analyses of peripheral blood cell samples from MDD patients reported mostly an overlap of the transcripts regulated by the glucocorticoid receptor activation in both men and women. But genetic variants acting on downstream epigenetic and regulatory elements were regulated in a sex-specific manner. This finding was correlated to the transcriptional signatures found in post-mortem brain tissue and the genome-wide association studies (GWAS) analyses showing an enrichment of these variant transcripts associated with MDD (Moore et al., 2021).

These results have been further expanded by the use of network-based approaches. Combined with conventional differential gene expression analyses, network-based approaches provide detailed data-driven molecular classifications associated with specific pathological states such as Alzheimer’s disease (Zhang et al., 2013), autism (Parikshak et al., 2013; Willsey et al., 2013), post-traumatic stress disorder (Breen et al., 2015), neurodegenerative diseases (Narayanan et al., 2014), stress in mice (Bagot et al., 2016, 2017; Labonté et al., 2017; Lorsch et al., 2018, 2019; Scarpa et al., 2020; Walker et al., 2022a), and MDD in humans (Labonté et al., 2017; Scarpa et al., 2020). This strategy revealed the existence of male and female MDD-specific gene networks modulating stress susceptibility in a sex-specific fashion *via* the activity of hub genes controlling distinct functional pathways. For instance, the authors identified the gene encoding for *DUSP6* in females and *EMX1* in males as drivers of stress susceptibility in a sex-specific fashion. The downregulation of *DUSP6* in the medial prefrontal cortex (mPFC) increased stress susceptibility while its overexpression rescued stress-induced depressive and anxiety-like behavioral deficits in females but not males (Labonté et al., 2017). This was associated with changes in the activity of the ERK intracellular signaling cascade and in the activity of pyramidal neurons in the mPFC of females but not males. Alternatively, the overexpression of *EMX1* in the mPFC increased depressive and anxiety-like behavioral responses in males but not females. This was also consistently associated with a potentiation of pyramidal neuron activity in a sex-specific fashion (Labonté et al., 2017). It should be emphasized that *DUSP6* was consistently downregulated in the mPFC of both women with MDD and stressed female mice after CVS. Additionally, an increased phosphorylation of ERK was found in females from both species in pyramidal neurons but not GABAergic interneurons. *DUSP6* downregulation in the mPFC, while increasing stress susceptibility, also reproduced a large proportion of the transcriptional changes observed in depressed and stressed females. Together, these findings highlight the contribution of *DUSP6* in the mPFC as a female-specific driver of stress susceptibility, and strongly supports the capacity of CVS to reproduce specific behavioral and molecular aspects of MDD in a sex-specific fashion.

Similar analyses with human cohorts also revealed a major sex difference in the expression of long non-coding RNAs (lncRNAs; Issler et al., 2020). Issler and colleagues recently revealed regulation of lncRNAs associated with depression in brain region and in a sex-specific fashion. Roughly 3% of differentially expressed lncRNA were commonly affected in men and women with MDD, similar to the levels reported above for protein coding genes (5%–10%; Labonté et al., 2017). The authors identified the primate-specific lncRNA *LINC00473* as a potential sex-specific mediator of depression in females specifically. The analyses revealed that this lncRNA was consistently downregulated across brain regions in women but not men with MDD, and its expression was strongly correlated with protein coding genes previously associated with MDD including *DUSP6*, *ARC*, *NR4A1*, *EGR1*, and *EGR2* (Orsetti et al., 2008; Covington et al., 2010; Li et al., 2015; Labonté et al., 2017). Interestingly, the downregulation of *LINC00473* in the mPFC was sufficient to rescue the social withdrawal induced by CSDS, and anxiety- and compulsive-like behaviors induced by CVS in females but not males (Issler et al., 2020). The authors further provided functional data suggesting that the pro-resilient effects induced by the downregulation of this lncRNA are associated with a reduction of the activity of pyramidal neurons (Issler et al., 2020). Interestingly, these effects are similar to what was reported with the downregulation of *DUP6* in females’ mPFC (Labonté et al., 2017), by impacting the activity of the CREB pathway known for its involvement in MDD (Carlezon et al., 2005). Whether these effects may be mediated by similar intracellular cascades or not, these results suggest that lncRNAs, while interacting with protein coding genes, are involved in the control of depressive- and anxiety-like behaviors in humans and mice.

More recently, insights into the transcriptional signatures associated with depressive traits and states have been made (Shukla et al., 2021). Using RNAseq from the anterior cingulate gyrus, Shukla and colleagues investigated transcriptional signatures from four different cohorts during: a first depressive episode; remission after the first episode; recurrent episodes, or remission after recurrent episodes. Interestingly, these analyses highlighted several patterns of differentially expressed genes, some of which showed consistent changes across every phase, but also robust patterns oscillating between episodes and remission phases. Importantly, only minimal overlap was found between genes found in the episode and remission phases. Further deconvolution analyses suggested that a cluster of genes co-expressing GABAergic markers such as SST, VIP (vasoactive intestinal peptide), and CRH (corticotrophin-releasing hormone) displayed phasic changes according to the disease states. This suggests that changes in interneuron function in the mPFC may be involved in the transition from state to trait phases in men’s MDD. Unfortunately, this study included only a limited number of samples from women, that prevented the authors to perform sex-specific analyses. It would be wrong to assume these findings are applicable to women with MDD, as transcriptional signatures from men and women with MDD differ. More work will be required to identify the transcriptional signatures defining state and trait MDD in the female brain. Nevertheless, these findings are consistent with previous studies performed in humans and mouse models of stress and support the alteration of the GABAergic signaling as a potential driver of depressive-like behaviors (Tripp et al., 2012; Soumier and Sibille, 2014; Hodes et al., 2015; Lin and Sibille, 2015; Shepard et al., 2016; Fee et al., 2017; Fuchs et al., 2017; Czéh et al., 2018; Shepard and Coutellier, 2018; Todorović et al., 2019; Girgenti et al., 2021). By dissociating transcriptional changes identified with depressive state and trait, these findings represent a significant step forward in the understanding of the molecular mechanisms underlying the expression and the consolidation of the disease.

### Transcriptional Studies in Mouse Models

It is interesting to note that a number of studies confirmed the capacity of different types of stress to reproduce a significant proportion of the molecular alterations associated with MDD in both sexes. For instance, consistent low transcriptional overlap has been reported in the mPFC and nucleus accumbens (NAc) of males and females after CVS (Hodes et al., 2015; Labonté et al., 2017). Several functional pathways have also been shown to be enriched with differentially expressed genes (DEG) in both human MDD and stressed males and females (Labonté et al., 2017; Scarpa et al., 2020). These changes result from alterations in the epigenetic regulation of gene expression that include modifications at the DNA methylation level. Indeed, the overexpression of the DNA methyltransferase 3 alpha (Dnmt3a) in the NAc was shown to increase stress susceptibility in both sexes while its downregulation made female mice resilient to 6 days of variable stress with no effect in males (Hodes et al., 2015). Interestingly, these behavioral effects were associated with significant transcriptional alterations distinctly affecting males and females. CVS was also shown to alter the regulation of microRNA (miRNA) expression discernably in males and females (Pfau et al., 2016). Previous analyses using RNAseq to screen miRNA profiles in males and females that underwent CVS revealed highly sex-specific signatures proposing that susceptibility and resilience to sCVS exhibited by males and females may result from a complex remodeling of miRNA signatures affecting coding genes. This was suggested for lncRNAs in human brains as well (Issler et al., 2020).

A similar reorganization of transcriptional structures was observed following CSDS (Bagot et al., 2016, 2017; Lorsch et al., 2018, 2019; Scarpa et al., 2020). In addition to what extent stress changes transcriptional profiles in the brain, these studies confirmed that resilience is a mechanism involving the activation of specific transcriptional programs required to elaborate and consolidate appropriate behavioral strategies to cope with stress. This was reported both at the differential expression and the gene network levels (Bagot et al., 2016, 2017; Lorsch et al., 2018, 2019; Scarpa et al., 2020), similar to what was observed in human MDD, but also after CVS and SI (Labonté et al., 2017; Seney et al., 2018; Scarpa et al., 2020). However, none of these studies included females, limiting their interpretation to males only. At the differential expression level, the number and identity of genes differentially expressed across brain regions were drastically different between males susceptible and resilient to CSDS (Bagot et al., 2016, 2017; Scarpa et al., 2020). The transcriptional organization of gene networks was also different between both phenotypes, with distinct gene networks being associated with the expression of stress susceptibility and resilience in males after CSDS. Importantly, the behavioral contribution of these gene networks was confirmed by a series of behavioral and functional studies. The susceptible-specific hub genes encoding for the Dickkopf Like Acrosomal Protein 1 (*Dkkl1*) and the neurogenic differentiation transcription factor 2 (*NeuroD2*), increased susceptibility to social stress, and induced behavioral despair and anxiety-like behaviors when overexpressed in the ventral hippocampus (vHPC) but not in the mPFC of male mice (Bagot et al., 2016). Overexpression of the gene sidekick cell adhesion molecule 1 (*Sdk1*), in the vHPC also promoted depressive and anxiety-like behavioral features to social stress. However, its overexpression in the mPFC induced pro-resilient effects in male mice (Bagot et al., 2016). Interestingly, the behavioral effects observed after the overexpression of these genes were associated with changes in neuronal activity in the vHPC. The overexpression of both *Dkk1l* and *Sdk1* increased spontaneous excitatory postsynaptic current frequency with no effect on amplitude (Bagot et al., 2016). Furthermore, the overexpression of these two hub genes induced a significant reorganization of the transcriptional structure of their respective gene networks in the vHPC (Bagot et al., 2016). Overall, this suggests that the regulation of specific hub genes promotes the expression of stress susceptibility by imposing functional changes in the activity of specific neuronal populations *via* a reorganization of its own network transcriptional structure. As these findings apply to males only, more work is needed to define the transcriptional profiles underlying the expression of susceptibility and resilience to social stress in female mice.

Further research on the transcriptional organization of gene networks in susceptible and resilient mice identified the *Esr1* gene, encoding for the estrogen receptor 1, as an upstream regulator that drives resilience to social stress in the NAc. The overexpression of *Esr1* in the NAc generated a robust pro-resilient phenotype in males exposed to CSDS and in females that experienced sCVS (Abelaira et al., 2013). These behavioral changes coincided with a consistent reorganization of transcriptional signatures. The authors noted a major overlap between transcriptional signatures from males after *Esr1* overexpression and resilient but not susceptible males after CSDS. In contrast, no significant overlap was observed between transcriptional signatures from males and females after *Esr1* overexpression, suggesting that the molecular mechanisms underlying the expression of resilience induced by *Esr1* may differ in males and females. It is also important to consider that *Esr1* may indeed be a driver of stress resilience but only in CSDS; classically, CVS in males and females does not induce the expression of a resilient phenotype. Further work is needed to address these important questions.

Lorsch et al. (2019) identified the transcription factor Zinc finger protein 189 (*Zfp189*) as an additional driver of resilience to social stress in the mPFC. The analyses revealed that *Zfp189* is one of the most connected key drivers within a resilient-specific gene network, and significantly upregulated in the mPFC of resilient mice after CSDS. Consistently, the human homolog *ZNF189* was significantly downregulated in the mPFC from MDD post-mortem tissue. Interestingly, the overexpression of* Zfp189* in the mPFC was shown to trigger pro-resilient responses when administered before stress exposure and rescued the susceptible phenotype when injected after exposure to CSDS consistent with a pro-resilient and antidepressant-like role for this key-driver. Further analyses confirmed that the pro-resilient effect of *Zfp189* was mediated by a specific reorganization of its own gene network, which is associated with resilience in the mPFC. More importantly, the authors showed that this effect was driven through direct interactions with CREB. Despite that *Zfp189* and its gene network have been identified in males, CREB knockdown (KO) induced the expression of a depressive-like phenotype to social stress in males and sCVS in females. The expression of *Zfp189* in CREB KO mice rescued these effects in both sexes (Lorsch et al., 2019). These results strongly support the role of Zfp189 as a driver of resilience to stress in both sexes, regardless of the type of stress used. Finally, the direct relationship of both proteins was shown through an elegant set of experiments that combined CRISPR gene editing with behavioral assessment. The authors used a specific strategy to specifically target CREB and *Zfp189* to either associate or segregate them in order to induce or prevent their physical interactions. Interestingly, targeting CREB to *Zfp189* *via* this approach increased resistance to social stress while creating a repressive environment around *Zfp189* gene loci. This decreased its expression in the mPFC and induced a pro-susceptible phenotype in male mice. Together these analyses provide substantial evidence for the role of *Zfp189* in mediating pro-resilient effects *via* a complex molecular cascade that involves direct interactions with CREB in the mPFC.

ELS has also been recently shown to induce different transcriptional changes across brain regions of males and females. This series of analyses was based on the two-hit stress model in mice: postnatal stress that occurs during postnatal days 10–20 increases susceptibility to social stress later in life (Peña et al., 2017, 2019). These behavioral effects have been associated with a series of transcriptional changes affecting several brain regions differently, including the ventral tegmental area (VTA), NAc, and mPFC in males and females, depending on the history of previous ELS. These analyses suggest that ELS primes molecular programs in different brain regions to be in a depressive-like state, thus being more plastic to a significant reorganization when challenged by additional stress during adulthood (Peña et al., 2017, 2019), or even drug abuse in a sex-specific fashion (Walker et al., 2022a). These findings led to the identification of specific genes as upstream regulators of transcriptional structures in these brain regions driving stress responses in a sex-specific fashion. While the genes encoding for alpha-synuclein (SNCA) and beta catenin (CTNNB1) were both predicted upstream regulators in female VTA and NAc, the orthodenticle homeobox 2 encoding gene, *Otx2*, was the highest-ranked upstream regulator of the pro-depressive transcriptional signature in males’ VTA (Peña et al., 2017, 2019). The functional and behavioral implication of *Otx2* as an upstream regulator of pro-depressive transcriptional signatures was further assessed by a series of behavioral experiments following its viral modification directly in the VTA. Transient *Otx2* overexpression in the VTA of juvenile male mice blocked susceptibility to adult social defeat and rescued the downregulation of several *Otx2* targets in this brain region (Peña et al., 2017). The transient juvenile suppression of *Otx2* expression in the VTA recapitulated the effects of postnatal stress on the expression of susceptibility to social stress during adulthood, which is associated with significant changes in the expression of its downstream target genes. It is important to note that these effects were specifically associated with the juvenile developmental period, as the overexpression of *Otx2* during adulthood only partially rescued behavioral and transcriptional effects, while its downregulation failed to induce behavioral susceptibility and changes in *Otx2* target gene expression (Peña et al., 2017).

Further analyses suggest that these effects may be mediated at least in part by epigenetic changes. Indeed, several targets of *Otx2* in the VTA were predicted to be enriched with the presence of the open chromatin mark H3K4me3 (Peña et al., 2017). Similar observations have been made concerning the epigenetic mechanisms mediating the effects of ELS in the NAc of males and females (Kronman et al., 2021). Kronman and colleagues showed that ELS induces a significant suppression of the repressive histone mark H3K79 specifically in males (Kronman et al., 2021). These effects were accompanied by cell-type-specific changes in the expression of the H3K79 writer and eraser, *Dot1l* and *Kdm2b*, respectively, in the NAc following a developmental trajectory. The expression of both genes was significantly increased in D2-expressing medium spiny neurons (MSN) of both males and females, an effect that was not seen early postnatally (PND21) but that became significant at a later developmental stage (PND35). This was maintained until adulthood, suggesting an incubation effect of ELS across developmental stages. Interestingly, *Dot1l* downregulation in D2-MSNs reversed the behavioral consequences of ELS-mediated behavioral susceptibility, while its overexpression in the same neuronal population replicated the behavioral phenotype induced by ELS in males, and to a lower extent in females. Conversely, the overexpression of *Kdm2b* in D2 expressing MSNs reversed ELS-induced behavioral phenotypes, whereas its downregulation increased stress susceptibility in males exclusively. As shown before with other key drivers and upstream regulators, the transcriptional profiles initiated by ELS were strikingly similar to those induced by *Dot1l* overexpression and inversed to *Dot1l* downregulation in D2 MSNs. Interestingly, further analyses were done to address the discrepancy between the upregulated expression of *Dot1l* and the downregulation of H3K79me2 in whole NAc after ELS. The results showed that the upregulation of *Dot1l* is associated with increased deposits of H3K79me2 at more genomic sites, but the loss of H3K79me2 found at a subset of sites is more important. This loss could be due to the coordinated induction of *Kdm2b* in the NAc.

### Interspecies Transcriptional Studies

Each of these studies provides valuable evidence that distinct mouse models are useful in testing the contribution of specific genes and transcriptional programs on behavioral responses to chronic stress. However, they still do not directly compare the extent of how they can accurately reproduce the transcriptional signatures relevant to MDD in the brain. This precise question was recently addressed by comparing the RNAseq transcriptional profiles generated from human post-mortem brain samples and three models of chronic stress including CVS, SI, and CSDS (Labonté et al., 2017; Scarpa et al., 2020). These analyses revealed a significant overlap between transcriptional alterations in the mPFC and NAc from human MDD and stressed mice, with each of the chronic stress paradigms capturing distinct aspects of MDD abnormalities. At the differential expression level, CVS and SI were shown to better reproduce the human conditions in the NAc and mPFC (Scarpa et al., 2020). It should be mentioned that these analyses have been done by controlling for the effect of sex. Indeed, not every dataset included females, and sex-specific analyses were not possible which limits the interpretation of these results. Nevertheless, these findings are consistent with previous comparative studies showing that both males and females that experienced CVS reproduce a significant proportion of the differential expression profiles observed in men and women with MDD (Labonté et al., 2017). These analyses also revealed a significant number of functional pathways that are enriched for DEGs in humans with MDD, and each of the different mouse models of stress. This suggests that the behavioral consequences of stress may be mediated by similar functional pathways in both species (Scarpa et al., 2020).

Importantly, network-based approaches provided similar conclusions. Consistent with previous studies (Tsaparas et al., 2006; Monaco et al., 2015; Eidsaa et al., 2017), all three mouse models were shown to share a significant level of co-expression structure in the mPFC and the NAc, although it is accepted that the human transcriptome acquired a certain complexity throughout evolution that is not shared in mouse (Pembroke et al., 2021). This approach identified gene networks sharing common co-expression structures associated with MDD and stress and enriched with genes differentially expressed in human and mouse models. For instance, the authors reported a gene network associated with the function and structure of oligodendrocytes (Scarpa et al., 2020). Interestingly, impaired myelin-related gene expression, along with reduced myelin thickness, have been reported in the cortex from suicide completers with a history of child abuse (Lutz et al., 2017; Tanti et al., 2018, 2021). Similarly, prolonged social isolation and social stress in mice have been shown to change oligodendrocyte gene expression that interferes with myelin integrity in the mPFC (Liu et al., 2012; Zhang et al., 2016). Amongst all the genes in this network, *Gab1* was identified as a hub gene preserved in humans with MDD and each of the three mouse models of chronic stress. *Gab1* is also known to enhance PI3K/AKT activation and to extend the duration of Ras/MAPK signaling (Kiyatkin et al., 2006). Additionally, it was shown to indirectly trigger myelination by increasing the expression of *Egr2* when activated by the protein kinase A (PKA; Ghidinelli et al., 2017). Altered oligodendrocyte function in MDD has also been supported by a recent study using single nuclei RNA sequencing to probe changes in gene expression across every cell type found in the mPFC of men with MDD (Nagy et al., 2020). Amongst all genes found differentially expressed, the majority were found in oligodendrocytes and a subpopulation of deep layer excitatory cells in the mPFC. Based on their predictions, the authors concluded that the relationship between these two clusters of cells could be explained in part by impairments in fibroblast growth factor signaling, steroid hormone receptor cycling, immune function, and cytoskeletal regulation, which could underly changes in mPFCsynaptic plasticity (Nagy et al., 2020). These results are also consistent with previous results showing metabolic, functional, and morphological changes in the mPFC with depression and chronic stress (Hare and Duman, 2020).

**Figure 2:**
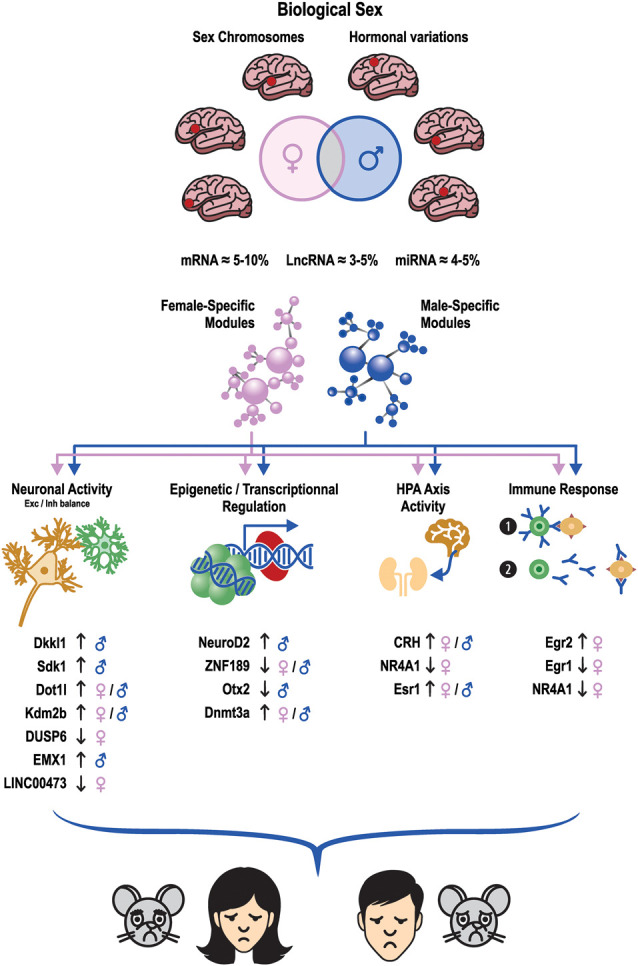
Males and females with MDD or stress share minimal transcriptional overlap across brain regions. These sex differences may be driven by biological factors such as sex chromosomes and hormonal variations. Similar differences are also observed at the gene network level where sex-specific transcriptional networks are associated with the expression of MDD in either males or females in humans but also with the expression of stress susceptibility or resilience in stressed male and female mice. These transcriptional changes interfere with the activity of several molecular, biological, and cellular processes such as neuronal activity, epigenetic and transcriptional regulation, the function of the HPA axis, and immune response. Ultimately, this leads to the expression of converging depressive-like behaviors in males and females sharing similar symptomatic and behavioral features. The orientation of the arrows next to listed genes indicates whether gene expression is upregulated or downregulated in the depressed/stressed conditions. mars: male symbol, ♀: female symbol.

Overall, these studies suggest that each mouse model can reproduce common but also unique molecular features relevant to the expression of the disease in humans with no unique model better than the others (Table 1; Figure 2). In other words, the decision for an appropriate model should be based not only on its capacity to reproduce certain behavioral aspects, but also its capacity to reproduce the transcriptional alterations relevant to the human condition. However, as female transcriptional data are not consistently available for each model, it is impossible to predict whether this capacity applies to both males and females. This cannot be simply addressed by directly overlapping human and mouse profiles, as important considerations such as gene orthology, correlation structures, and connectivity need to be taken when comparing the transcriptional structures of two different species. Additional clinical variables such as age, hormonal status, and pathological comorbidities that are difficult to account for in human post-mortem studies are also important considerations when performing interspecies sex-specific studies. Nevertheless, based on previous findings from human and mouse studies (Labonté et al., 2017; Lorsch et al., 2018, 2019; Scarpa et al., 2020), it is tempting to speculate that both males and females would reproduce specific aspects of the human condition, but most likely not the same. More work will be required to address this important question and consolidate the benefits of using mouse models to study specific molecular mechanisms underlying the expression of MDD in both sexes.

**Table 1:** Summary of recent transcriptomic analyses done by RNA-sequencing characterizing transcriptional profiles in human MDD post-mortem brains and different animal models of depressive-like behaviors.

**Transcriptional studies in humans from post-mortem tissues:**						
**Human sex samples (M/W)**	**Sample size**	**Region of Interest**	**Main Findings**	**Studies**
M & W	26 MDD (13 M & 13 W) and 22 Ctrl (13 M & 9 W); A cohort of 32 M (15 MDD & 17 Ctrl); A cohort of 18 W (6 MDD & 12 Ctrl)	vmPFC, OFC, dlPFC, aINS, NAc, vSUB	Low transcriptional overlap and divergent gene network structures between males and females across brain regions	Labonté et al. (2017)
M & W	50 MDD (26 M & 26 controls, 24 W & 24 Ctrl)	dlPFC, sgACC, BLA	Low transcriptional overlap between males and females across brain regions	Seney et al. (2018)
M & W	143 samples from 46 Ctrl (26 M & 20 W), 52 PTSD (26 M & 26 W), and 45 MDD (27 M & 18 W)	PFC, AMY, HIPP, dlPFC	Divergent transcriptomic signatures between PTSD and MDD. Low transcriptional overlap between males and females	Girgenti et al. (2021)
M & W; adolescents & children (M & W)	Cohort 1: 289 samples from 93 W (48 MDD & 45 Ctrl) and 1960 M (81 MDD & 115 Ctrl); Cohort 2: 584 children and adolescents with 350 MDD & 234 Ctrl; Cohort 3: 1774 samples from 879 MDD & 756 Ctrl	Blood samples analysis associated with six brains regions of interest. Only a significant result with BA25/ACC is presented	High overlap of the GR transcripts between sexes with only an enrichment of the eQTL in females	Moore et al. (2021)
M & W	Cohort of 50 MDD & Ctrl	OFC, dlPFC, vmPFC, NAc, aINS, vSUB	*LINC00473* is a sex-specific mediator of depression in females specifically	Issler et al. (2020)
M & W with few proportions of W	90 samples (20 Ctrl, 20 MDD, 15 in remission after one episode, 20 in recurrent episodes & 15 remissions after recurrent episodes)	dlPFC/ACC	Changes in interneurons function in the mPFCare involved in the transition from state to trait in MDD	Shukla et al. (2021)
M & W	78 samples (27 MDD suicided with CA, 25 without CA & 26 Ctrl)	ACC	CA induces epigenetic reprogramming of myelin in adults	Lutz et al. (2017)
M & W	36 samples (18 MDD with CA & 18 MDD without CA)	vmPFC	Long-term changes in connectivity related to imbalance of oligodendrocytes and myelin remodeling in MDD patients with CA	Tanti et al. (2018)
M & W	11 Ctrl from 9 M & 2 W, 26 MDD without CA from 14 M & 12 W, 12 MDD with CA from 9 M & 3 W	vmPFC/BA11-12	Decreased neuroplasticity of cortical circuits through the enhancement of developmental OPC-mediated PNN formation in MDD patients with CA	Tanti et al. (2021)
M	34 samples (17 MDD & 17 Ctrl)	dlPFC	Significant differential expression of oligodendrocytes associated with dysregulation of excitatory neurons in MDD	Nagy et al. (2020)
**Transcriptional studies in mouse models:**						
**Animals**	**Models**	**Sample size**	**Age**	**Region of Interest**	**Main Findings**	**Studies**
C57BL/6J M & F mice	CVS	40 mice (10 M/groups, & 10 F/groups)	8 weeks	vmPFC and NAc	*DUSP6* and *EMX1* are drivers of stress susceptibility in a sex-specific manner	Labonté et al. (2017)
C57BL/6J M & F mice	CVS	3-5 mice/groups	8 weeks	OFC, dlPFC, vmPFC, NAc, aINS, vSUB	LncRNA *LINC00473* is a sex-specific mediator of depression in females specifically	Issler et al. (2020)
C57BL/6J M & F mice	sCVS	48 (4 mice/library & 3 libraries/sex/stress condition)	8-12 weeks	NAc	Low overlap between transcriptional profiles in the NAc and PFC in stressed males and females	Hodes et al. (2015)
C57BL/6J M & F mice	sCVS	60 (5 mice/library & 3 libraries/sex/stress condition)	8 weeks	NAc	Little overlap of the transcriptional and post-transcriptional profiles between sexes	Pfau et al. (2016)
C57BL/6J M mice	CSDS	12 (4 mice/library & 3 libraries/sex/stress condition)	8 weeks	vHIP, PFC, NAc, AMY	Overexpression of two specific hub genes induced a significant reorganization of the transcriptional structure of their respective gene networks in the vHIP	Bagot et al. (2016)
C57BL/6J mice	CSDS	10 Ctrl, 8 resilient, 14 non-responders to treatments (8 + 6), 6 susceptible, 12 responders to treatments (6 + 6)		PFC, NAc, HIP, AMY	Transition from susceptible to resilient transcriptional profiles following pharmacological treatments	Bagot et al. (2017)
C57BL/6J mice	CSDS (M), CVS (F)	27 mice (6-8M mice/groups & 6-7 F mice/groups)	8 weeks	NAc, PFC	Estrogen receptor 1 is an upstream regulator that drives resilience to social stress	Lorsch et al. (2018)
C57BL/6J M & F mice	CSDS (M) & sCVS (F)	10 mice (5/groups)	8 weeks	PFC, vHIP, BLA, NAc	*Zfp189* is a hub gene driving resilience to social stress	Lorsch et al. (2019)
C57BL/6J M & F mice	ELS (MS and limited nesting) alone or followed by STVS or CSDS	4-6 mice/groups 5-6 mice/groups	Adult mice	VTA, NAc, PFC	ELS primes molecular programs toward a reorganization when challenged by stress during adulthood	Peña et al., 2019
C57BL/6J M mice	2-hit stress model, CSDS	3 mice/groups/sex	Adult and adolescent mice	VTA	*Otx2* overexpression rescued depressive-like behaviors and reversed *Otx2*-targets gene expression	Peña et al. (2017)
C57BL/6J M & F mice	CSDS, ELS	2 mice/groups/sex	10-12 weeks for CSDS	NAc	ELS induces a sex and cell type specific reorganization of H3K79 profiles	Kronman et al. (2021)
**Interspecies transcriptional studies:**						
**Subjects**	**Models**	**Age**	**Sample size**	**Region of Interest**	**Main Findings**	**Studies**
C57BL/6J mice; M & F humans	CSDS, SI, CVS	MDD: 45+/–17 years old & Ctrl: 48+/–17	26 MDD (13 M & 13 W), 22 Ctrl (13 M & 9 W); 10 CVS mice/sex; 30 SI M & 15 M Ctrl; 11 M CSDS/phenotypes	PFC & NAc	CVS, SI and CSDS reproduce common but also unique transcriptional changes relevant to the expression of MDD	Scarpa et al. (2020)

*Abbreviations: M, men/males; W, women; F, females; PTSD, post-traumatic stress disorder; MDD, major depressive disorder; Ctrl, controls; CA, child abuse; eQTL, cis-expression quantitative trait loci; LncRNA, long non-coding RNA; sCVS, subchronic variable stress; CVS, chronic variable stress; CSDS, chronic social defeat stress; ELS, early life stress; MS, maternal separation; STVS, subthreshold variable stress; SI, social isolation; OPC, oligodendrocytes progenitor cells; PNN, perineuronal nets; vmPFC, ventromedial prefrontal cortex; OFC, orbitofrontal cortex; dlPFC, dorsolateral prefrontal cortex; aINS, anterior insula; NAc, nucleus accumbens; vSUB, ventral subiculum; sgACC, subgenual anterior cingulate cortex; AMY, amygdala; HIPP, hippocampus; mPFC, medial prefrontal cortex; vmPFC, ventromedial prefrontal cortex; vHIP, ventral hippocampus; PFC, prefrontal cortex; BLA, basolateral amygdala; VTA, ventral tegmental area; BA, Brodmann area; ACC, anterior cingulate cortex*.

## Conclusion

Fundamental research using animals is an absolute necessity to improve our understanding of complex human conditions. Here, we have reviewed the strengths and weaknesses of some of the most widely used models to study the molecular and functional impact of chronic stress on the expression of depressive and anxiety-like behaviors. Amongst the multiple conclusions that can be drawn, no unique model can fully reproduce the human condition. Indeed, the clinical manifestation of the disease varies between individuals either qualitatively or quantitatively (Soderlund and Lindskog, 2018) which cannot be accounted for in animals. Several complex behavioral features and traits related to the disease cannot be evaluated without falling into anthropomorphic considerations. Furthermore, the clinical representation of the disease keeps evolving throughout the pathological process (Zahn-Waxler et al., 2000). Thus, rather than mimicking MDD and anxiety in mice as a whole, one should consider modeling specific aspects of the disease that can be accurately reproduced and quantified in mice and more importantly differently in each mouse model.

Nevertheless, data strongly support the use of animal models to study the molecular mechanisms underlying the expression of stress susceptibility and resilience in both males and females, although only a few studies properly integrated females in their analysis. As of now, studies investigating the transcriptional programs underlying the expression of MDD and anxiety in humans have revealed drastic differences between men and women. This should be considered carefully since the lack of overlap in DEG between stressed males and females should not always be interpreted as a sign of sex differences (Mukamel, 2022). With the development of novel approaches combining the assessment of differential expression profiles with transcriptional overlap, gene ontology and gene network-based approaches integrating correlation structures and connectivity measures, the sum of converging evidence is strongly supporting the existence of true sex differences in the transcriptional organization of gene networks across the brain that may drive the expression of behavioral alterations in a sex-specific fashion (Labonté et al., 2017; Lorsch et al., 2018, 2019; Seney et al., 2018; Walker et al., 2022a, b).

Most importantly, the transcriptional signatures associated with each type of stress share common core features but also unique aspects relevant to the human condition. In this sense, types of stress with psychosocial constructions affect the brain transcriptome differently than other stress types relying on physical paradigms. In perspective, this is in line with our understanding of how environmental challenges are impacting brain activity through epigenetic mechanisms (Fatma and Labonté, 2019) and adds to the importance of considering not only the behavioral features but also the molecular systems affected by different types of stress when choosing an appropriate mouse model. Ultimately, this choice may have a crucial impact on behavioral, morphological, functional, and molecular findings. For instance, transcriptional alterations that increase the activity of mPFC neurons have been shown to promote stress susceptibility in animals undergoing CVS (Labonté et al., 2017; Issler et al., 2020) while changes that induce similar functional impacts on mPFC activity have been associated with resilience and anti-depressant properties in the CSDS model (Bagot et al., 2016). Similarly, certain transcriptional changes triggering stress susceptibility in females induce no effect in males and the opposite has also been shown (Labonté et al., 2017).

Probably the most important remaining question is what are the mechanisms underlying these differences either at the behavioral or transcriptional levels. Amongst the different potential players, sex chromosomes and gonadal hormones come to mind. Both X and Y chromosomes contain genes encoding for different chromatin writers and erasers as well as several transcription factors (Sene et al., 2013; Seney et al., 2013; Dossat et al., 2017). These genes are crucially involved in various developmental processes and are likely to be impacted differently by environmental factors and ultimately by hormonal influences (Puralewski et al., 2016; Jaric et al., 2019). Similarly, molecular processes and emotional responses are also importantly regulated by gonadal hormones which broaden the contribution of sex-specific biological correlates underlying stress responses in males and females (Bangasser and Cuarenta, 2021; Bhargava et al., 2021; Rainville et al., 2022). More recently, FCG mice were used to dissect the behavioral and transcriptional impact of gonadal hormones and sex chromosomes over stress responses in males and females (Paden et al., 2020). Interestingly, results show that XX male carriers recapitulate XX females’ behavioral profiles. Similar findings were also reported for XY female carriers and XY males. At the transcriptional level, 25% of the differences between males and females were related to sex chromosomal influences while 23%–31% of these differences were associated with gonadal hormones (Paden et al., 2020). Interestingly, despite the extent of the transcriptional differences, the authors reported that a large proportion of the transcriptional changes in males and females were in fact clustered on similar functional pathways (Paden et al., 2020). This is very similar to the findings reported in human post-mortem tissue (Labonté et al., 2017; Seney et al., 2018; Girgenti et al., 2021) and supports the idea that common functional pathways may be impacted in males and females with MDD but *via* different genes. However, the contribution that sex chromosomes and gonadal hormones have, especially during crucial developmental phases, remains unknown and more work will be required to fully understand the complex interplay between sex chromosomes, gonadal hormones, and transcriptional programs in controlling the development of emotional responses in stressed males and females (Paden et al., 2020; Seney and Logan, 2021).

Overall, this suggests that several transcriptional programs are in place to control neuronal activity and brain function and these programs are affected distinctly by different types of stress in males and females. As of now, only the tip of the iceberg has been revealed and much more work is needed to provide a better understanding of the molecular mechanisms underlying stress susceptibility and resilience in males and females. While work in human populations is crucial to drive this initiative, animal models remain one of the best strategies to provide mechanistic insights into the effects. With this in mind, future work should consider using these approaches to reveal the transcriptional signatures underlying specific symptomatic profiles in humans. With the knowledge that each of the models can accurately reproduce specific behavioral and molecular aspects of MDD and anxiety in males and females, such initiatives should provide interesting insights into the systems to target more precisely in order to treat specific symptoms, rather than the complex syndrome.

## Author Contributions

MT and BL reviewed the litterature and wrote the manuscript. All authors contributed to the article and approved the submitted version.

## Conflict of Interest

The authors declare that the research was conducted in the absence of any commercial or financial relationships that could be construed as a potential conflict of interest.

## Publisher’s Note

All claims expressed in this article are solely those of the authors and do not necessarily represent those of their affiliated organizations, or those of the publisher, the editors and the reviewers. Any product that may be evaluated in this article, or claim that may be made by its manufacturer, is not guaranteed or endorsed by the publisher.
